# Supplementary material for: Magnetically Labelled iPSC‐Derived Extracellular Vesicles Enable MRI/MPI‐Guided Regenerative Therapy for Myocardial Infarction
Source: J Extracell Vesicles. 2025 Oct 8;14(10):e70178. doi: 10.1002/jev2.70178 (PMC12508251; doi:10.1002/jev2.70178)
Supplement: Supplementary file 1 — Supplementary Material: jev270178‐sup‐0001‐SuppMat.pdf [file JEV2-14-e70178-s001.pdf]

Supporting Information

Magnetically Labeled iPSC-Derived Extracellular Vesicles Enable MRI/MPI-Guided Regenerative Therapy for Myocardial Infarction

Wenshen Wang[1,2], Zheng Han[1,2,3], Safiya Aafreen[1,4], Cristina Zivko[5,6], Olesia Gololobova[7], Zhiliang Wei[1,2], Geoffrey Cotin[8], Delphine Felder-Flesc[8], Vasiliki Mahairaki[5,6], Kenneth W. Witwer[7], Jeff W.M. Bulte[1,2,4,9,10], Robert G. Weiss[2,11], Guanshu Liu[1,2,10]

1. F.M. Kirby Research Center, Kennedy Krieger Institute, Baltimore, MD, USA
2. Russell H. Morgan Department of Radiology and Radiological Science, Division of MR Research, Johns Hopkins University School of Medicine, Baltimore, MD, USA
3. Department of Biomedical Engineering, University of Central Oklahoma, Edmond, OK, USA
4. Department of Biomedical Engineering, Johns Hopkins University School of Medicine, Baltimore, MD, USA
5. Department of Genetic Medicine, Johns Hopkins University School of Medicine, Baltimore, MD, USA
6. The Richman Family Precision Medicine Center of Excellence in Alzheimer's Disease, Johns Hopkins School of Medicine, Baltimore, MD, USA
7. Department of Molecular and Comparative Pathobiology, Johns Hopkins University School of Medicine, Baltimore, MD, USA
8. SUPERBRANCHE SAS, Strasbourg, France
9. Department of Chemical & Biomolecular Engineering, Johns Hopkins University Whiting School of Engineering, Baltimore, MD, USA
10. Department of Oncology, Johns Hopkins University School of Medicine, Baltimore, MD, USA
11. Department of Medicine, Division of Cardiology, Johns Hopkins University School of Medicine, Baltimore, MS, USA

* Corresponding author:
Guanshu Liu, Ph.D.
707 N. Broadway, Baltimore, MD, USA 21205
Phone (office): 443-923-9500; Fax: 410-614-3147
Email: guanshu@mri.jhu.edu

S1. Cell culture of iPSCs and quality control (QC)

iPSCs were thawed and passaged onto vitronectin-coated 6-wells plates (250,000 cells/well) in complete Essential 8™ medium (E8, Gibco, 2 mL/well) supplemented with 10 µM of Y-27632 ROCK inhibitor (Ri, STEMCELL Technologies), as previously described[1,2]. The E8 medium without Ri was replaced daily. Cells were passaged every 3 days upon reaching ~80-90% confluency (**Figure S1**). They were cultured for 10-12 passages.

Cultured cells were monitored daily for morphological characteristics to ensure the maintenance of healthy, undifferentiated colonies. Regular quality control assessments were performed to ensure the integrity and pluripotency of the iPSC line. Genomic stability was evaluated using G-band karyotyping and single nucleotide polymorphism (SNP) arrays. Pluripotency markers Oct4 and Tra-1-60 were assessed by immunohistochemistry (IC). Functional pluripotency was validated through trilineage differentiation assays. Cells were routinely checked for mycoplasma contamination.

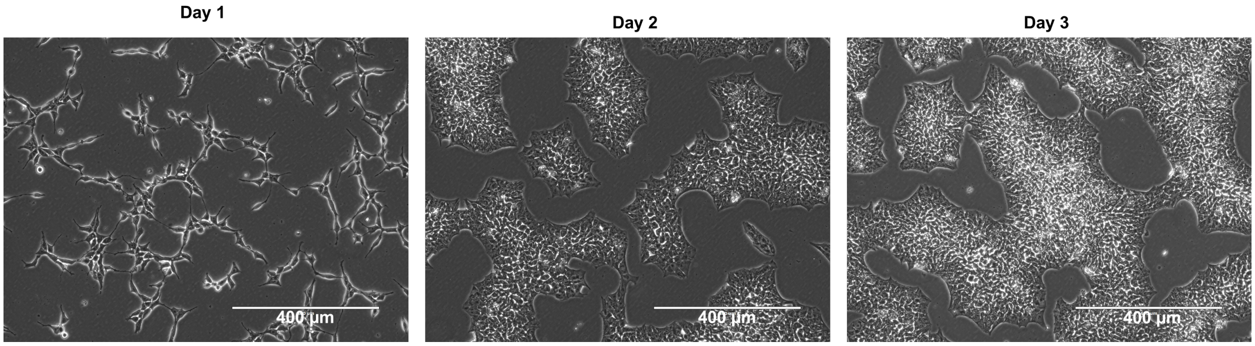

Figure S1. **Representative brightfield images of the BC1-iPSCs in culture.**

S2. Western blots for EV characterization

iPSC-EVs and magneto-iPSC-EVs samples (18 µL) were lysed in 1x radioimmunoprecipitation assay buffer (RIPA, Cell Signaling Technology,Cat. #9806) for overnight (approx. 18 hours) at 4°C. Lysates were heated at 95 °C for 5 minutes together with 6.67 µL 4x Laemmli Sample Buffer (Bio-Rad, #1610747; non-reducing condition). Lysates were resolved using a 4% -15% Criterion TGX Stain-Free Precast gel (Bio-Rad, # 5678084), with Spectra Multicolor Broad Range protein ladder (Thermo Scientific, # 26634). Stain-free images of a gel were obtained

using Bio-Rad Gel Doc imager. Proteins were then transferred onto a PVDF membrane (Invitrogen, # IB24001) using iBlot 2 semi-dry transfer system (Invitrogen). Blots were first probed using primary antibodies, including CD9 (1:1000, Ms, BioLegend #312102), Calnexin (1:1000, Rb, Abcam #22595), in PBST (PBS with 0.05% Tween-20 (BioXtra, #P7949) and 5% Blotting Grade Blocker (Bio-Rad, #1706404). Then, SuperSignal West Pico PLUS Chemiluminescent Substrate (Thermo Scientific, # 34580) was applied to the membrane, and blots were imaged using an iBright 1500FL Imager (Thermo Fisher). A representative Western Blot image is provided in **Figure S2**, showing the presence of EV-positive marker CD9 and absence of EV-negative marker Calnexin.

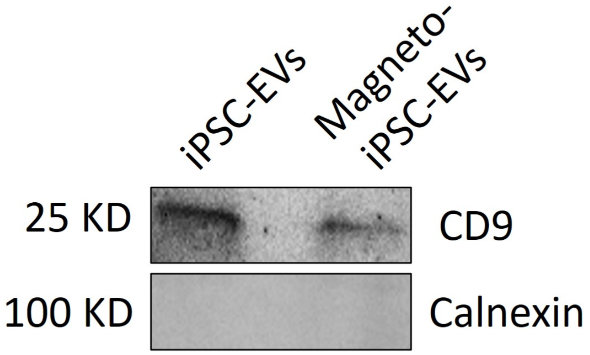

Figure S2. Representative western blot of iPSC-EVs and magneto- iPSC-EVs.

S3. Quantitative RT-PCR

Primer sequences used in qRT-PCR are listed in **Table S1**.

Table S1. Primer sequences used for RT-PCR

Gene	Primers	
	Forward	Reverse
TGF-β [3]	GGCCAGATCCTGTCCAAGC	GTGGGTTTCCACCATTAGCAC
Arg-1 [4]	GACCGTTGTGTGTGTTCTGG	GATGAGCAGCATCACAAGGA
IL–6 [5]	CTGCAAGAGACTTCCATCCAGTT	AGGGAAGGCCGTGGTTGT
18S [6]	GTAACCCGTTGAACCCCATT	CCATCCAATCGGTAGTAGCG
Mcp1 [7]	CCACTCACCTGCTGCTACTCA	TGGTGATCCTCTTGTAGCTCTCC

S4. Characterization of EVs after electroporation

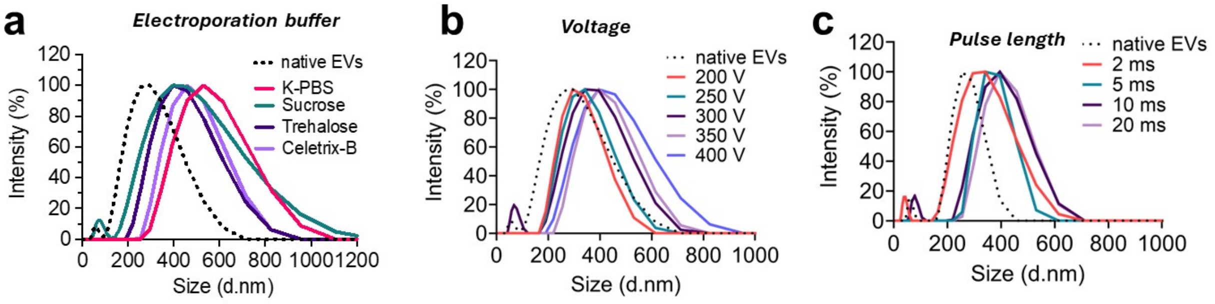

Figure S3. Effect of (**a**) buffer (pulse fixed at 400 V/10 ms), (**b**) pulse voltage (fixed pulse duration =10 ms), and (**c**) pulse length (fixed pulse voltage = 300 V) on the size of iPSC-EVs after electroporation as revealed by DLS measurements. The result shows that electroporation conducted in trehalose-containing KPBS buffer resulted in the least size increase (435.0±6.1 nm), followed by Celetrix buffer (477.0±24.9 nm), sucrose-containing KPBS (488.9±73.8 nm), and KPBS (584.9±61.1 nm), as compared to native EVs (293.4±29.9 nm).

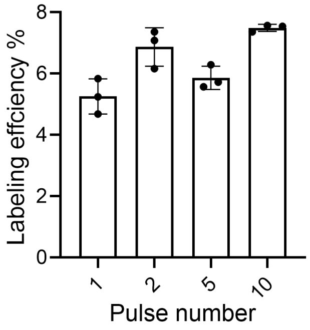

Figure S4. Effect of pulse number on magnetic labeling efficiency in iPSC-EVs. Electroporation pulses were applied at 300 V with the total duration kept at 10 ms.

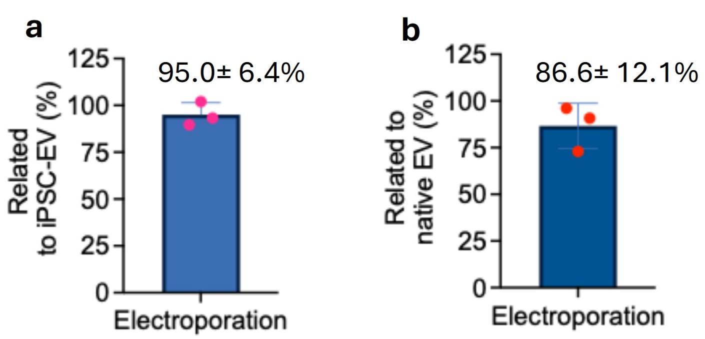

Figure S5. Characterization of cargo loss (percentage) of (**a**) Protein and (**b**) RNA contents in iPSC-EVs caused by the optimized electroporation (300 V, 10 ms, and 2 pulses).

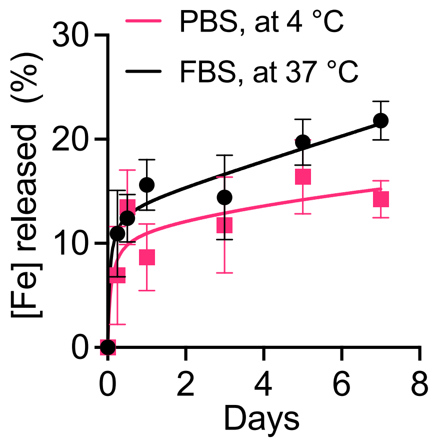

Figure S6. Stability of magnetic labeling as characterized by the released [Fe] from magneto-iPSC-EVs after incubating PBS (room temperature) or serum (FBS, 37 °C) at a volume ratio of 1: 2 for up to 7 days.

S5. Small RNA-sequencing analysis

The top 20 abundant miRNAs in iPSC-EVs/magneto-iPSC-EVs identified by small RNA-seq are summarized in **Table S2**, along with their functions.

Table S2. Summary of highly expressed miRNA in iPSC-EVs/magneto-iPSC-EVs.

Order of abundance	**Gene name**	**Function (reference)**
1	hsa-miR-148a-3p	Anti-inflammation [8]
2	hsa-miR-21-5p	Anti-apoptosis and anti-inflammation [9,10]
3	hsa-miR-92a-3p	Anti-apoptosis [11]
4	hsa-miR-151a-3p	Anti-apoptosis [12]
6	hsa-let-7f-5p	Anti-apoptosis and anti-inflammation [13,14]
8	hsa-miR-34c-5p	Anti-apoptosis and anti-inflammation [15]
9	hsa-let-7i-5p	Anti-apoptosis and anti-inflammation [16-18]

11	hsa-let-7g-5p	Anti-apoptosis and anti-inflammation [19,20]
13	hsa-miR-30d-5p	Anti-apoptosis and anti-inflammation* [21]
14	hsa-miR-143-3p	Anti-apoptosis [22]
15	hsa-miR-26a-5p	Anti-apoptosis and anti-inflammation* [23]
16	hsa-let-7a-5p	Anti-fibrotic and anti-inflammation [24]
17	hsa-miR-423-3p	Anti-apoptosis* [25]
19	hsa-miR-27b-3p	Anti-apoptosis and anti-inflammation [26]
20	hsa-miR-3184-3p	Anti-apoptosis and anti-inflammation [27]

* Previously demonstrated for myocardial infarction treatment.

S6. In Vitro Functional Characterization of Magneto-iPSC-EVs

Cell Culture and Preparation

Human umbilical vein endothelial cells (HUVECs), human dermal fibroblasts (hDFs), rat cardiomyoblasts (H9c2), and murine macrophages (RAW264.7) were maintained according to standard protocols. For HUVEC-based assays, wells were pre-coated with 10 µg/mL human fibronectin (Sigma, F1141) in phosphate-buffered saline (PBS) for 30 minutes at 37°C prior to cell seeding.

EV Labeling and Quantification

iPSC-derived EVs were fluorescently labeled using MemGlow™ 640 (Cytoskeleton, MG04) by incubating with 1 µM dye for 1 hour at room temperature in darkness. Unbound dye was removed using Vesi-SEC-micro size-exclusion columns (Vesiculab). Labeled EVs were quantified by NTA and adjusted to working concentrations in either serum-free medium.

Cellular Uptake Assay

Cells were seeded in 96-well plates in triplicate at densities calculated to achieve approximately 70% confluence within 24 hours. Fluorescently labeled EVs were diluted to 1×10^9 particles/mL, and 100 µL (1×10^8 particles) was added to each well. Cells were incubated with labeled EVs for 6 hours at 37°C in 5% CO_2. During the final 10 minutes, Hoechst 33342 (1 µg/mL final

concentration) was added for nuclear staining. Cells were then fixed with 4% paraformaldehyde (PFA) for 15 minutes, washed twice with PBS, and imaged using fluorescence microscopy.

HUVEC Tube Formation Assay

Matrigel (Corning, BD 356230) was thawed overnight on ice and 50 µL was added to pre-chilled 96-well plates, followed by polymerization at 37°C for 45 minutes. HUVECs (1×10^5 cells/mL) were prepared in EV-depleted EGM-2 medium (Lonza, CC-3162) and treated as follows: (1) untreated control, (2) VEGF (10 ng/mL; Cell Signaling Technology, 48143S), (3) iPSC-EVs (2×10^8 particles), (4) magneto-iPSC-EVs (2×10^8 particles), and (5) SuperSPIO-His nanoparticles (0.34 µg/well). Cell suspensions (100 µL) were carefully seeded onto Matrigel-coated wells and incubated for 6 hours at 37°C in 5% CO_2. Cells were then stained with Calcein AM (2 µM; Invitrogen, C1430) and Hoechst 33342 (1 µg/mL) for 20 minutes before imaging.

HUVEC Migration Scratch Assay

HUVECs were seeded at 5×10^4 cells per well in fibronectin-coated 24-well plates and cultured overnight in complete EGM-2 medium until reaching 100% confluence. Uniform scratches were created using sterile 200 µL pipette tips, and wells were gently washed with PBS to remove detached cells. Medium was replaced with basal EGM-2 containing EV-depleted FBS and supplemented with: (1) iPSC-EVs (2×10^8 particles/well), (2) magneto-iPSC-EVs (2×10^8 particles/well), or (3) SuperSPIO-His nanoparticles (1 µg/well). Brightfield images were captured at predefined positions immediately after scratching (0 h) and at 8 h and 24 h post-treatment using consistent imaging parameters.

Anti-Fibrotic Assay

hDFs were seeded at 6,000 cells per well in 96-well plates using FGM-2 medium (Lonza, CC-3132) and incubated overnight for attachment. Medium was replaced with FGM-2 containing EV-depleted FBS and the following treatments: (1) PBS control, (2) TGF-β alone (2 ng/mL; Bio-Rad, PHP143B), (3) iPSC-EVs + TGF-β (2×10^8 particles), (4) magneto-iPSC-EVs + TGF-β (2×10^8 particles), and (5) SuperSPIO-His + TGF-β (0.34 µg/well). After 48-hour incubation, cells were fixed with 4% PFA for 15 minutes, permeabilized with 0.1% Triton X-100 for 10 minutes, and blocked with 5% BSA for 30 minutes. Primary antibody incubation was performed using Alexa Fluor 488-conjugated anti-α-SMA antibody (1:200; BioLegend, 614853) for 1 hour at room

temperature. Nuclei were counterstained with Hoechst 33342 (1 µg/mL) for 10 minutes.

Imaging and Analysis

Fluorescence images were acquired using a Leica Thunder imaging system with DFC9000 GT camera. Migration assay images were captured using the EVOS™ FL Color Imaging System (Thermo Scientific, AMEFC4300). Angiogenic parameters were quantified using the Angiogenesis Analyzer plugin in ImageJ. Wound closure was calculated as the percentage reduction in wound width relative to time 0. All experiments were performed in triplicate with at least three independent biological replicates.

Results

Cellular Uptake of Magneto-iPSC-EVs

As shown in **Fig. S7**, both iPSC-EVs and magneto-iPSC-EVs demonstrated efficient cellular uptake across all tested cell types (H9c2 cardiomyoblasts, RAW264.7 macrophages, hDFs, and HUVECs) after 6-hour incubation. Fluorescence microscopy revealed intracellular localization of MemGlow640-labeled EVs, with comparable uptake efficiency between conventional and magnetic EVs, suggesting that magnetic labeling does not impair cellular internalization. Among all cells studied, the selectivity of cell uptake is determined to be RAW264.7 > HUVECs > H9c2 > hDFs.

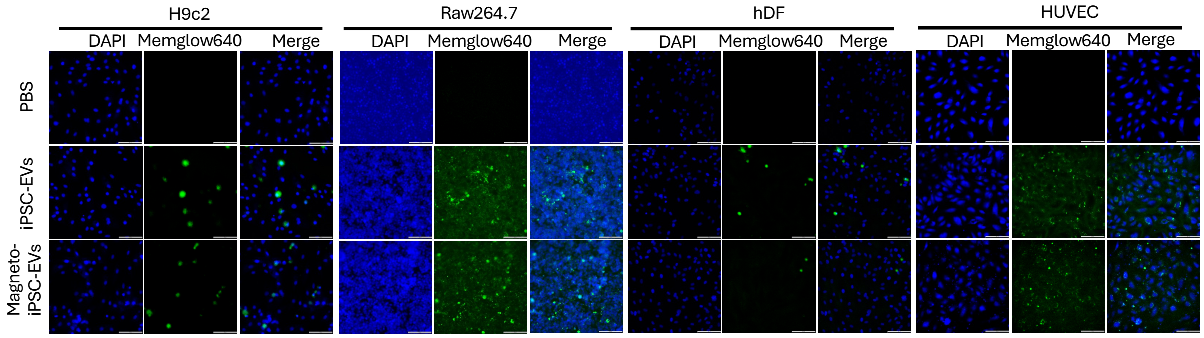

Figure S7. Uptake of MemGlow640-labeled iPSC-EVs and Magneto-iPSC-EVs by various cell types (H9c2, Raw264.7, hDF, and HUVECs) after 6 h incubation. Representative fluorescence images show MemGlow640 signal (green) and Hoechst (blue).

Enhanced Angiogenic Properties

HUVEC tube formation assays (**Fig. S8**) revealed that both iPSC-EVs and magneto-iPSC-EVs

significantly promoted angiogenesis compared to controls (both SPIO and PBS groups). Quantitative analysis showed increased numbers of junctions and branches in EV-treated groups, with magneto-iPSC-EVs demonstrating comparable or enhanced angiogenic potential relative to VEGF-positive controls. SuperSPIO-His nanoparticles alone showed minimal angiogenic activity.

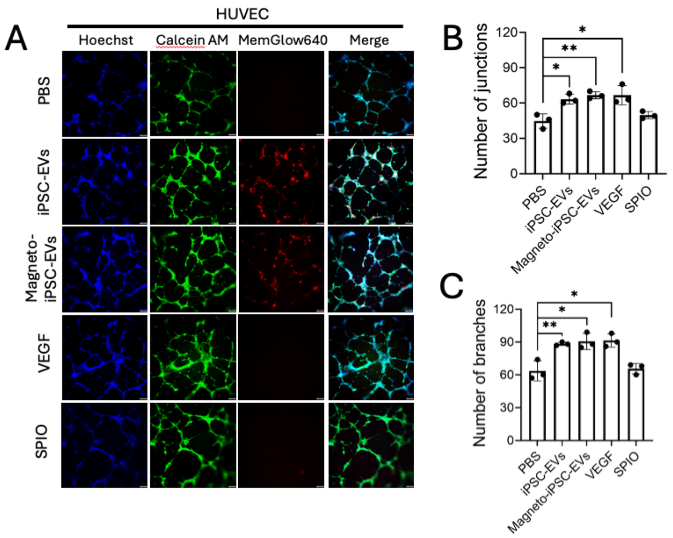

Figure S8. (A) Representative fluorescent microscopy images of HUVECs after treatment with PBS, iPSC-EVs, magneto-iPSC-EVs, VEGF and SPIO for 6 h. MemGlow640-labeled EVs (red) colocalize with Calcein AM-labeled live cells (green). VEGF was used as a positive control. (B, C) Quantification of angiogenic parameters using the Angiogenesis Analyzer tool in ImageJ: number of junctions (B) and number of branches (C).

<u>Accelerated Wound Healing</u>

Scratch assay analysis demonstrated that both EV formulations significantly accelerated HUVEC migration and wound closure. At 24 hours post-treatment, magneto-iPSC-EVs achieved comparable wound closure compared to conventional iPSC-EVs, suggesting enhanced regenerative capacity. Quantitative measurements showed progressive reduction in wound width over time in EV-treated groups.

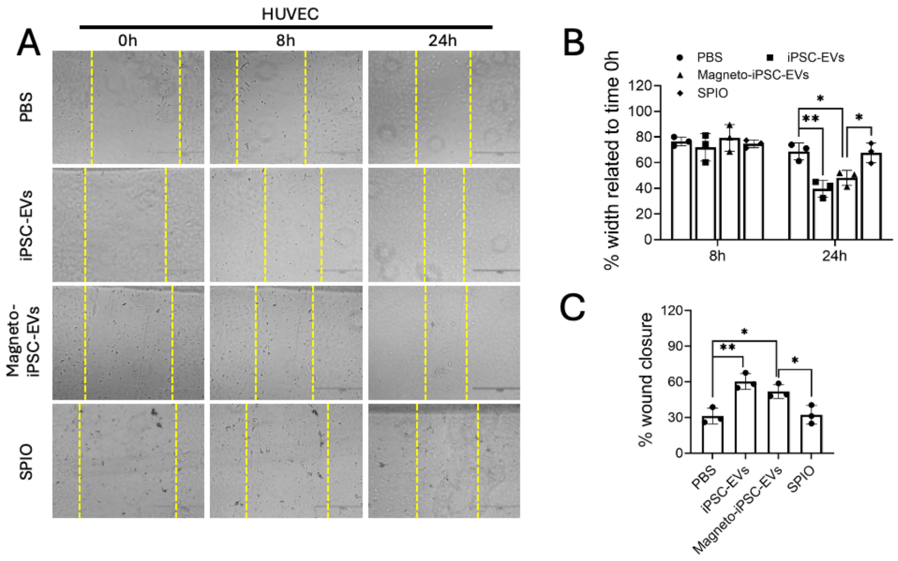

Figure S9. (A) Representative images of HUVEC scratch wound healing assays at 0, 8, and 24 h post-treatment. Yellow dashed lines indicate the edge of the wound area. (B) Quantification of wound width (normalized to 0 h) at 8 h and 24 h. (C) Percent wound closure at 24 h.

<u>Anti-Fibrotic Effects</u>

TGF-β treatment induced robust α-SMA expression in hDFs, characteristic of myofibroblast activation and fibrotic transformation. Both iPSC-EVs and magneto-iPSC-EVs substantially attenuated TGF-β-induced α-SMA upregulation, demonstrating significant anti-fibrotic properties. The magnetic EVs showed comparable or enhanced anti-fibrotic effects relative to conventional EVs.

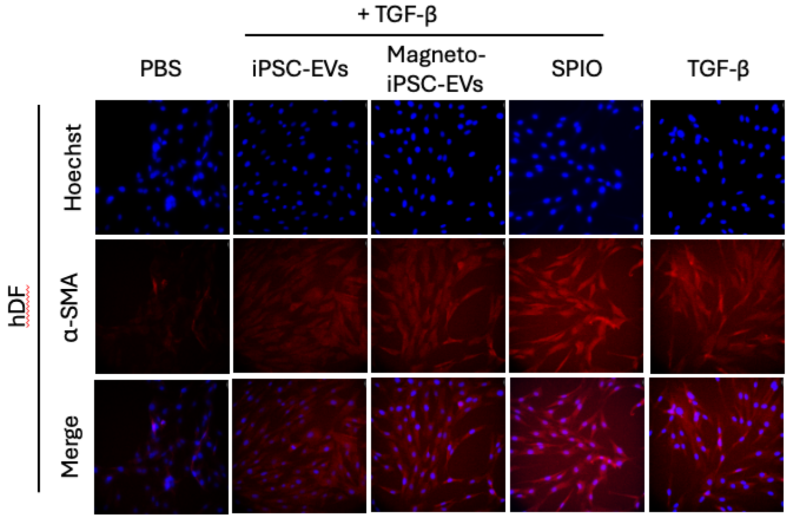

Figure S10. Representative images showing that iPSC-EVs and magneto-iPSC-EVs substantially reduce the TGF-β-induced fibrosis in hDFs.

S7. Ischemia-reperfusion injury (IRI) mouse model

The cardiac ischemia-reperfusion injury (IRI) model was performed according to previously published protocols[28]. In brief, mice were anesthetized using 3%–4% isoflurane for induction and 0.03–0.07 mg/kg buprenorphine (subcutaneous) for pre-operative analgesia. Anesthesia was maintained with 1%–2% isoflurane and 2 mg/kg succinylcholine (intraperitoneal). The mice were intubated, mechanically ventilated, and maintained at a constant body temperature throughout the procedure.

A left thoracotomy was performed to expose the heart, and the left anterior descending (LAD) coronary artery was occluded for 35 minutes using a 7-0 PROLENE suture and PE10 tubing. The chest cavity was temporarily closed during the occlusion period. Five minutes before the end of occlusion, the chest was reopened, and the suture was removed to allow reperfusion. After closure of the chest, a second dose of buprenorphine (0.06–0.075 mg/kg, subcutaneous) was administered for post-operative analgesia. Sham-operated mice underwent the same surgical procedures without LAD ligation.

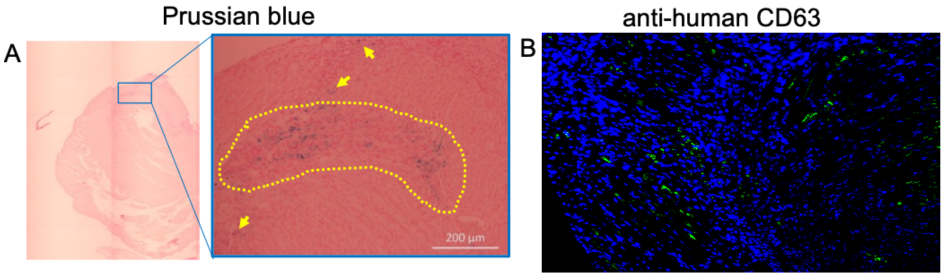

Figure S11. Confirming the presence of EVs after 7 days using human EV-specific marker staining. A) Prussian blue staining showing stain show the accumulation of iron after 7 days of intramyocardial injection of magneto EVs. B) Anti-human CD63 staining of approximately the same location of the heart revealed the presence of human-sourced EVs in the IRI heart. Note that the Prussian blue and CD63 staining were performed on adjacent tissue sections, rather than on the same slice.

Supplementary References

1. Chou, B.K., *et al.* Efficient human iPS cell derivation by a non-integrating plasmid from blood cells with unique epigenetic and gene expression signatures. *Cell Res* **21**, 518-529 (2011).
2. Sagar, R., *et al.* Generation and Characterization of a Human-Derived and Induced Pluripotent Stem Cell (iPSC) Line from an Alzheimer's Disease Patient with Neuropsychiatric Symptoms. *Biomedicines* **11**(2023).
3. Kang, J.I., *et al.* p62-Induced Cancer-Associated Fibroblast Activation via the Nrf2-ATF6 Pathway Promotes Lung Tumorigenesis. *Cancers (Basel)* **13**(2021).
4. Choo, Y.W., *et al.* M1 Macrophage-Derived Nanovesicles Potentiate the Anticancer Efficacy of Immune Checkpoint Inhibitors. *Acs Nano* **12**, 8977-8993 (2018).
5. Chen, F., Guo, N., Cao, G., Zhou, J. & Yuan, Z. Molecular analysis of curcumin-induced polarization of murine RAW264.7 macrophages. *J Cardiovasc Pharmacol* **63**, 544-552 (2014).
6. Turner, N., *et al.* A selective inhibitor of ceramide synthase 1 reveals a novel role in fat metabolism. *Nat Commun* **9**, 3165 (2018).
7. Lin, S., *et al.* Targeting parvalbumin promotes M2 macrophage polarization and energy expenditure in mice. *Nat Commun* **13**, 3301 (2022).
8. Anastasio, C., Donisi, I., Colloca, A., D'Onofrio, N. & Balestrieri, M.L. MiR-148a-3p/SIRT7 Axis Relieves Inflammatory-Induced Endothelial Dysfunction. *Int J Mol Sci* **25**(2024).
9. Xue, J., *et al.* miR-21-5p inhibits inflammation injuries in LPS-treated H9c2 cells by regulating PDCD4. *Am J Transl Res* **13**, 11450-11460 (2021).
10. Lv, X., Liang, J. & Wang, Z. MiR-21-5p reduces apoptosis and inflammation in rats with spinal cord injury through PI3K/AKT pathway. *Panminerva Med* **66**, 256-265 (2024).
11. Niu, H., *et al.* miR-92a is a critical regulator of the apoptosis pathway in glioblastoma with inverse expression of BCL2L11. *Oncol Rep* **28**, 1771-1777 (2012).
12. Li, C., *et al.* Microglia-Derived Exosomal microRNA-151-3p Enhances Functional Healing After Spinal Cord Injury by Attenuating Neuronal Apoptosis via Regulating the p53/p21/CDK1 Signaling Pathway. *Front Cell Dev Biol* **9**, 783017 (2021).
13. Han, L., *et al.* MicroRNA Let-7f-5p Promotes Bone Marrow Mesenchymal Stem Cells

Survival by Targeting Caspase-3 in Alzheimer Disease Model. *Front Neurosci* **12**, 333 (2018).

14. Xu, L., Song, Q., Ouyang, Z., Zhang, X. & Zhang, C. let7f-5p attenuates inflammatory injury in in vitro pneumonia models by targeting MAPK6. *Mol Med Rep* **23**(2021).
15. Tu, Y. & Hu, Y. MiRNA-34c-5p protects against cerebral ischemia/reperfusion injury: involvement of anti-apoptotic and anti-inflammatory activities. *Metab Brain Dis* **36**, 1341-1351 (2021).
16. Thomas, J.J.*, et al.* MiR-451a and let-7i-5p loaded extracellular vesicles attenuate heme-induced inflammation in hiPSC-derived endothelial cells. *Front Immunol* **13**, 1082414 (2022).
17. Wang, X.*, et al.* MicroRNA Let-7i negatively regulates cardiac inflammation and fibrosis. *Hypertension* **66**, 776-785 (2015).
18. Zhang, J.*, et al.* Overexpression of Exosomal Cardioprotective miRNAs Mitigates Hypoxia-Induced H9c2 Cells Apoptosis. *Int J Mol Sci* **18**(2017).
19. Xiao, Y.C.*, et al.* The Peripheral Circulating Exosomal microRNAs Related to Central Inflammation in Chronic Heart Failure. *J Cardiovasc Transl Res* **15**, 500-513 (2022).
20. Wu, M., Gao, Y. & Chen, B. Mechanism of acteoside-activated let-7g-5P attenuating Abeta-induced increased permeability and apoptosis of brain microvascular endothelial cells based on experimental and network pharmacology. *Neuroreport* **33**, 714-722 (2022).
21. Boxhammer, E.*, et al.* MicroRNA-30d-5p-A Potential New Therapeutic Target for Prevention of Ischemic Cardiomyopathy after Myocardial Infarction. *Cells* **12**(2023).
22. Yang, Z., Wang, J., Pan, Z. & Zhang, Y. miR-143-3p regulates cell proliferation and apoptosis by targeting IGF1R and IGFBP5 and regulating the Ras/p38 MAPK signaling pathway in rheumatoid arthritis. *Exp Ther Med* **15**, 3781-3790 (2018).
23. Wen, X.*, et al.* Effect of miR-26a-5p targeting ADAM17 gene on apoptosis, inflammatory factors and oxidative stress response of myocardial cells in hypoxic model. *J Bioenerg Biomembr* **52**, 83-92 (2020).
24. Chen, S.Y.*, et al.* Engineered extracellular vesicles carrying let-7a-5p for alleviating inflammation in acute lung injury. *J Biomed Sci* **31**, 30 (2024).
25. Yang, T.R.*, et al.* Resina draconis inhibits the endoplasmic-reticulum-induced apoptosis of myocardial cells via regulating miR-423-3p/ERK signaling pathway in a tree shrew

myocardial ischemia- reperfusion model. *J Biosci* **44**(2019).

26. Li, W.*, et al.* miR-27b-3p, miR-181a-1-3p, and miR-326-5p are involved in the inhibition of macrophage activation in chronic liver injury. *J Mol Med (Berl)* **95**, 1091-1105 (2017).

27. Xu, H.*, et al.* miR-3184-3p enriched in cerebrospinal fluid exosomes contributes to progression of glioma and promotes M2-like macrophage polarization. *Cancer Sci* **113**, 2668-2680 (2022).

28. Naumova, A.V.*, et al.* Xanthine oxidase inhibitors improve energetics and function after infarction in failing mouse hearts. *Am J Physiol Heart Circ Physiol* **290**, H837-843 (2006).
